# Examining Artificial Intelligence Chatbots’ Responses in Providing Human Papillomavirus Vaccine Information for Young Adults: Qualitative Content Analysis

**DOI:** 10.2196/79720

**Published:** 2026-02-18

**Authors:** Alfu Laily, Laura M Schwab-Reese, Megan Davish, Emily Cahue, Kathryn J LaRoche, Natalia M Rodriguez, Robert J Duncan, Randolph D Hubach, Monica L Kasting

**Affiliations:** 1 Department of Public Health Purdue University West Lafayette, IN United States; 2 Cancer Prevention and Control Program Indiana University Simon Comprehensive Cancer Center Indianapolis, IN United States; 3 Human Development and Family Studies Colorado State University Fort Collins, CO United States

**Keywords:** artificial intelligence, health communication, papillomavirus vaccines, large language models, qualitative research

## Abstract

**Background:**

The growing use of artificial intelligence (AI) chatbots for seeking health-related information is concerning, as they were not originally developed for delivering medical guidance. The quality of AI chatbots’ responses relies heavily on their training data and is often limited in medical contexts due to their lack of specific training data in medical literature. Findings on the quality of AI chatbot responses related to health are mixed. Some studies showed the quality surpassed physicians’ responses, while others revealed occasional major errors and low readability. This study addresses a critical gap by examining the performance of various AI chatbots in a complex, misinformation-rich environment.

**Objective:**

This study examined AI chatbots' responses to human papillomavirus (HPV)–related questions by analyzing structure, linguistic features, information accuracy and currency, and vaccination stance.

**Methods:**

We conducted a qualitative content analysis following the approach outlined by Schreier to examine 4 selected AI chatbots’ (ChatGPT 4, Claude 3.7 Sonnet, DeepSeek V3, and Docus [General AI Doctor]) responses to HPV vaccine questions. These questions, simulated by young adults, were adapted from items on the Vaccine Conspiracy Beliefs Scale and Google Trends. The selection criteria for AI chatbots included popularity, accessibility, countries of origin, response update methods, and intended use. Two researchers, simulating a 22-year-old man or woman, collected 8 conversations between February 22 and 28, 2025. We used a deductive approach to develop initial code groups, then an inductive approach to generate codes. The responses were analyzed based on a comprehensive codebook, with codes examining response structure, linguistic features, information accuracy and currency, and vaccination stance. We also assessed readability using the Flesch-Kincaid Grade Level and Reading Ease Score.

**Results:**

All AI chatbots cited evidence-based sources from reputable health organizations. We found no fabricated information or inaccuracies in numerical data. For complex questions, all AI chatbots appropriately deferred to health care professionals’ suggestions. All AI chatbots maintained a neutral or provaccine stance, corresponding with scientific consensus. The mean and range of response lengths varied [word count; ChatGPT: 436.4 (218-954); Claude: 188.0 (138-255); DeepSeek: 510.0 (325-735); and Docus: 159.4 (61-200)], as did readability [Flesch-Kincaid Grade Level; ChatGPT: 10.7 (6.0-14.9); Claude: 13.2 (7.7-17.8); DeepSeek: 11.3 (7.0-14.7); and Docus: 12.2 (8.9-15.5); and Flesch-Kincaid Reading Ease Score; ChatGPT: 46.8 (25.4-72.2); Claude: 32.5 (6.3-67.3); DeepSeek: 43.7 (22.8-67.4); and Docus: 40.5 (19.6-58.2)]. ChatGPT and Claude offered personalized responses, while DeepSeek and Docus lacked this. Occasionally, some responses included broken or irrelevant links and medical jargon.

**Conclusions:**

Amidst an online environment saturated with misinformation, AI chatbots have the potential to serve as an alternative source of accurate HPV-related information to conventional online platforms (websites and social media). Improvements in readability, personalization, and link accuracy are still needed. Furthermore, we recommend that users treat AI chatbots as complements, not replacements, to health care professionals’ guidance on clinical settings.

## Introduction

Globally, human papillomavirus (HPV) was responsible for an estimated 620,000 new cancer cases in women and 70,000 in men in 2019 [1]. In the United States, HPV causes roughly 48,000 new cancer cases annually and is the most common sexually transmitted infection [2,3]. Although effective vaccines are available [4], uptake among eligible adults remains suboptimal. In 2022, only 57.7% of eligible women and 34.8% of eligible men (ages 19-26 years) had received more than one dose of the HPV vaccine [5]. Adequate knowledge about HPV vaccination plays a critical role in promoting vaccine uptake among eligible adults [6].

The internet has become a widely used resource for people seeking information on health-related topics, including the HPV vaccine [7,8]. Information about the HPV vaccine is a popular online search topic in the United States, with Google Trends indicating an annual search traffic growth of 8.6% from 2010 to 2021 [8]. Research has also demonstrated that HPV vaccine communication uses specific search terms (eg, #HPV and #HPVVaccine) that are prevalent on online social media platforms such as Instagram [9,10], YouTube [11], and Twitter (now known as X) [12]. While these online platforms serve as a powerful tool for disseminating health information, they also provide a potential space for inaccurate information to spread, including inadvertently false information (misinformation) and deliberate falsehoods (disinformation) [13,14]. On Instagram, a primarily visual platform, approximately 55.8% of postings regarding HPV were provaccine, whereas 42.2% had an antivaccine perspective [9]. Additionally, the hashtag #*Gardasil* on Instagram has been used to circulate conspiracy theories and unsupported and false claims of HPV vaccine-related injuries [10]. YouTube, a popular video-centric social media space, users-generated content regarding the HPV vaccine showed a greater number of likes in videos with a negative tone compared to those with a positive tone [11]. On X, a platform for brief text-based posts, content containing erroneous information regarding the HPV vaccine were 5.44 times more likely to be shared than educative content [12], indicating that inaccurate posts received higher audience engagement. Given these trends, the World Health Organization (WHO) has identified the “uncontrolled dissemination of misinformation,” particularly on the topic of vaccination, as one of the most pressing health challenges for the coming decade [15].

The quality of online information related to the HPV vaccine could potentially influence vaccine decision-making [16]. Individuals who seek health information online showed lower acceptance of the HPV vaccination, which may be associated with discouraging information found online [17]. Furthermore, the overwhelming amount of information available online can lead to doubt and anxiety, making it challenging for individuals to make informed health decisions [18]. The abundance of information, much of which contains inaccurate information, may also contribute to low levels of HPV knowledge, as literature indicated that only 36.1% of young adults in the United States were aware that HPV causes more cancers than just cervical cancer [19]. This is concerning because knowledge of HPV correlates positively with HPV vaccination intention and uptake [20-22].

In recent years, there has been a growing inclination toward using internet-based artificial intelligence (AI) chatbots for accessing health-related information, including topics related to sexual health [23-26]. AI chatbots are digital systems created to mimic conversations with humans through text or voice, and their application in health care is rapidly expanding, ranging from answering general patient questions about sexual health to supporting medication adherence [25,27]. Large language models (LLMs) are AI tools built on multilayer recurrent neural networks and trained on extensive datasets of natural language to produce text that resembles human language [28]. AI chatbots are powered by LLMs, which are trained to generate human-like responses [29]. This generative ability relies on deep learning techniques, systems that mimic how the human brain handles information using neural networks to find patterns in data [30-32]. Examples of currently available AI chatbots based on deep learning include ChatGPT and DeepSeek [30,31,33], which analyze user input and then offer intelligent, contextually relevant responses for general purposes. In contrast, Docus is specifically designed to analyze health information, offer tailored health management, and provide laboratory test interpretations to facilitate health-related decision-making [34].

AI chatbots like ChatGPT are inevitably being used in support of health care settings, even though they were not originally intended for such purposes [35]. This is partly attributed to the user-friendly nature of LLMs used in the AI chatbot, making it convenient for individuals seeking health information [36]. Additionally, young adults aged <25 years and those identifying as technologically savvy had higher levels of AI chatbot acceptability [27,37]. However, it is important to note that the accuracy of AI chatbots’ responses, which partly depends on the quality of their training data, is compromised in medical contexts due to the lack of specific training data in medical literature [35,38]. The inability of AI chatbots to clarify their decision-making process presents challenges in identifying and rectifying potential biases or errors [35,39,40].

Several studies have revealed complex and often contradictory findings regarding the quality of AI chatbots' responses on health-related topics. A study demonstrated that ChatGPT produced higher-quality responses than those provided by physicians from patients’ questions pooled from a social media forum [41]. A specific knowledge-based chatbot trained exclusively on HPV vaccine information outperformed general-purpose generative pretrained transformer models in both accuracy and relevance [42]. In tasks involving physician-generated medical questions, responses generated from ChatGPT 3.5 and ChatGPT 4 were rated as “nearly all correct” or “completely correct” 50% of the time [43]. However, for these, the median accuracy scores exceeded mean scores, suggesting the presence of occasional but substantial wrong answers [43]. Bing Copilot’s responses to queries about prescription medications in the United States were found to be difficult to read, as indicated by low Flesch Reading Ease Scores, raising concerns about its readability and accessibility [44]. Although prior studies have evaluated AI chatbot responses, they typically focused on a single system, using physician-generated questions or purpose-built chatbots, which may not capture the full range of real-world interactions of various AI chatbots from the perspective of general public users.

While AI chatbots stand out as a remarkable technological feat, their application in providing health information raises concerns regarding the perpetuation of misinformation or inaccurate recommendations and therefore requires attention. To address this, the primary aim of this study is to investigate how 4 selected AI chatbots respond to HPV vaccine-related questions, with wording modified to reflect the language commonly used by young adults (22 years). The secondary aim of the study was to examine the content’s structure and patterns, linguistic features, information accuracy, information currency, and the AI chatbots’ stance on HPV vaccination, whether supportive, neutral, or contrary. This study distinguishes itself by conducting a qualitative content analysis of a diverse sample of both general-purpose and health-specific AI chatbots on HPV-related topics. By using questions tailored to the language of young adults, it provides a more ecologically valid assessment of AI chatbots’ performance and their utility in the real world.

## Methods

### Question Set

We created semistructured questions adapted from the Vaccine Conspiracy Beliefs Scale (VCBS) items [45] and Google Trends query [46] as prompts asked in AI chatbots. The original VCBS consists of seven statements: (1) vaccine safety data are often fabricated, (2) immunizing children is harmful and this fact is covered up, (3) pharmaceutical companies cover up the dangers of vaccines, (4) people are deceived about vaccine efficacy, (5) vaccine efficacy data are often fabricated, (6) people are deceived about vaccine safety, and (7) the government is trying to cover up the link between vaccines and autism. We modified the original VCBS scripts to reflect language commonly used by young adults and tailored the content to focus specifically on the HPV vaccine (Multimedia Appendix 1). We also used Google Trends [46], a publicly accessible web service, to capture search-term volume for the Google search engine over time. In order to investigate potential questions regarding the HPV vaccine (search term: HPV vaccine) during the past 12 months (from January 1, 2024, to January 1, 2025). Google Trends recorded both top queries (the most popular or “evergreen” inquiries within the specified search parameters, maintaining relative consistency over time) and rising queries (the inquiries with the most substantial increase in search frequency compared to the previous time period, signifying a surge in relative interest) [46]. We identified HPV-related search terms from both top and rising queries and rephrased them into questions (Multimedia Appendix 1). Finally, 2 researchers (EC and MD) within the young adult age group reviewed the question set and provided feedback to ensure clarity and relevance to their communication style.

### Sample and Data Collection

We collected 8 conversations (2 each from ChatGPT 4, Claude 3.7 Sonnet, DeepSeek V3, and Docus [General AI Doctor]) of AI chatbots’ responses to an HPV-related question set. The versions used for ChatGPT, Claude, and DeepSeek were the latest available without a paid subscription at the time of testing, while Docus was tested using the latest version available with a paid subscription. Our researchers started the “chat” by introducing users’ assigned sex at birth (male or female) and age (22 years old) in order to simulate a young adult population. Supervised by female (LMS-R, KJL, NMR, and MLK) and male (RJD and RDH) faculty members, all of whom have PhDs, 3 trained female public health graduate students (AL, MD, and EC) gathered 2 conversations from each AI chatbot, using different IP addresses, on different days, and with different sex enactments, for a total of 8 conversations from all AI chatbots. We copied and pasted those responses into a word processor and then uploaded them into HyperRESEARCH 4.5.4 (Researchware, Inc), a qualitative data analysis software. Since no human participants were involved, we did not establish a relationship prior to the commencement of the study nor created field notes. Furthermore, we only followed our predefined questions set, without introducing any off-topic or unrelated prompts into the AI chatbots during the “chat.” No one else was present during data collection, except for the research team members. The duration of each collection ranged from 14 minutes to 27 hours and 5 minutes; one AI chatbot's unpaid subscription enabled a wait time after inputting several prompts, and there were server errors that prolonged the data collection process.

### Rationale for Selecting the Reviewed AI Chatbots

The selected AI chatbots represented a diverse range of popularity, accessibility, country of origin, response update method, and intended use. ChatGPT had the highest global daily visits, while DeepSeek and Claude had lower visits [47]. ChatGPT was chosen primarily because it has the highest user traffic, making it likely that a significant number of young adults also engage with the platform. Due to the sudden surge in popularity of DeepSeek during the study period, as well as its origin in China, DeepSeek was selected for inclusion. Other than Docus (General AI Doctor), users could access reviewed AI chatbots without a subscription. ChatGPT and Claude were based in the United States, DeepSeek originated from China, and Docus originated from Armenia. We distinguished AI chatbots’ update methods between dynamic and static by examining whether each AI chatbot accessed real-time updated online information (dynamic) or relied on fixed, pretrained knowledge (static). We assessed the update methods by asking each AI chatbot, “What is the most recent information you can access?” ChatGPT responded with the current date (February 5, 2025), indicating dynamic updates, while Claude (October 2024), DeepSeek (June 2024), and Docus (October 2023) provided fixed cutoff dates, showing they rely on static knowledge. Other than Docus, which was developed specifically to provide health-related consultations, other AI chatbots were developed for general purposes (Table 1). Therefore, to add nuances to our study, Docus was included in this study because of its specific model.

**Table 1:** Artificial intelligence chatbots' characteristics for sample selection.

	ChatGPT 4	Claude 3.7 Sonnet	DeepSeek V3	Docus (General AI Doctor)
Global daily visit [47]	100-150 million	<15 million	<50 million	—^a^
Access	Free	Free	Free	Paid subscription
Originated from	United States	United States	China	Armenia
Information update methods	Dynamic	Static	Static	Static
Purpose	General	General	General	Health consultation

^a^Not available.

### Data Analysis

We conducted a qualitative content analysis following the approach outlined by Schreier [48]. Initially, 3 researchers with qualitative methods training (AL, EC, and MD) thoroughly reviewed all AI chatbot responses to gain a general understanding of the data. During this phase, we identified preliminary ideas that could serve as potential code groups and codes. We adopted a deductive, concept-driven approach to develop initial code groups based on our research objectives and question set. Within these groups, we applied an inductive, data-driven approach to generate codes based on all AI chatbot responses. The code-building process involved the use of subsumption and progressive summarizing strategies. As we reviewed the responses, we identified a distinct idea and checked whether an existing code captured this idea. If the idea was already represented, we ensured the code was grouped under the appropriate code group. If not, we created a new code to reflect the previously uncaptured idea. This iterative process continued until no new ideas emerged, thereby automatically meeting the criteria of saturation.

After generating initial code drafts, 3 researchers (AL, EC, and MD) collaborated to compile code groups and codes into a single comprehensive codebook (Multimedia Appendix 2). We reviewed the codebook to ensure that each code was mutually exclusive, and the codebook was collectively exhaustive to represent our data. A faculty member (LMS-R) provided feedback on the codebook before its application. To enhance consistency in code application, researchers (AL, EC, and MD) met and pilot-coded a subset of the data. Due to the straightforward and descriptive nature of the codebook, no coding disagreements arose. During code application, 2 independent researchers double-coded each AI chatbot response. Any discrepancies in coding were discussed and resolved.

We analyzed AI chatbot responses based on structure and patterns, linguistic features, accuracy, information currency, and stance on the HPV vaccination. Structure and pattern captured the structural and content-related elements of responses, including organization format (eg, paragraphs, bullet points, and numbered lists), logical reasoning and relevance of information to the question, details and length, and how the response concluded (eg, with a summary, follow-up question, or suggested next steps). Linguistic features included the use specific terminology, use of user-centric (referencing users’ age or sex) and emotional validation sentences (acknowledging user concerns and feeling or providing reassurance), use of language to convey statistics (eg, numeric vs relative terms), and assessed readability using the Flesch-Kincaid Grade Level and Reading Ease Score to determine suitability for lay audiences [49]. The Flesch-Kincaid Grade Level indicates the educational level required to understand a text passage, ranging from fifth grade (level 5) to college graduate (level 12+) [50,51]. The Flesch-Kincaid Reading Ease Score measures how easy it is to read a text passage, with scores ranging from 0 to 100 and higher scores indicating greater ease [52]. We adapted cutoff scores of ≤8 for the Flesch-Kincaid Grade Level and ≥50 for the Reading Ease Score to determine the readability for lay audiences [49]. Accuracy was assessed based on factual claims and numerical data in the AI chatbot responses, considering their alignment with current scientific evidence and HPV vaccine guidelines, including source credibility from reputable organizations (eg, the Centers for Disease Control and Prevention [CDC] and WHO) or peer-reviewed journals. Sources cited or links included in responses were manually checked to verify their credibility and to ensure they directed users to the information referenced in the AI chatbot’s response. Our research team consists of experts in sexual health (AL [53,54], KJL [55,56], RDH [57,58], and MLK [59]), health communication (AL [60,61], LMS-R [62,63], RDH [64], and MLK [65]), LLM and AI (LMS-R [66,67]), and HPV vaccination (AL [54], NMR [68], and MLK [69]), ensuring content accuracy. Content was considered current if information or fact sheets were published within 12 months or studies published within 5 years from data collection. HPV vaccination stance was assessed from the response to each question and categorized as pro, neutral, or contra.

The Flesch-Kincaid scores, how the response concluded, and vaccination stance were also assessed from each AI chatbot’s response to a question. However, we did not assess Flesch-Kincaid scores for responses containing links to avoid potential skewed results. Since each response set included 21 to 24 questions, this resulted in a corresponding range of 21 to 24 data points per set for conclusion style and vaccination stance, while Flesch-Kincaid scores had a range of 9 to 21 data points for Grade Level and Reading Ease scores.

### Ethical Considerations

As no human subjects were involved, ethics board reviews were not required. Furthermore, this study adheres to the COREQ (Consolidated Criteria for Reporting Qualitative Research) checklist. Specifically, our Methods section includes information from domain 1 (research team and reflexivity), domain 2 (study design), and parts of domain 3 (analysis and findings). The remainder of domain 3 is reported in our Results section.

## Results

We collected the 8 conversations between February 22 and 28, 2025, using 21-24 questions modified from VCBS items [45] and Google Trends queries [46]. Numbers following AI chatbot names (eg, Claude_1 and Claude_2) indicate dataset identifiers used to distinguish different responses from the same AI chatbots.

### Structure and Pattern

In structure and pattern of the responses, we observed organization format, logical reasoning, information relevance, details and length, and conclusion styles of each response.

#### Organization

In terms of the sentence organization format when answering one question, all AI chatbots responded more often using bulleted or numbered lists, or a combination of both, instead of paragraph-style responses. ChatGPT more frequently used bulleted points nested under a numbered list than other formatting styles. Claude more often used bulleted points but sometimes responded using only numbered lists or bullet points under numbered lists. DeepSeek typically used an organization of bulleted points under a numbered list, and at times used bulleted points nested within other bulleted points under a numbered list:

1. For Individuals Aged 9-14 Years

- Number of Doses: 2 doses
- Schedule:
First Dose: Given at the start of the series. Second Dose: Given 6-12 months after the first dose.

This age group has a stronger immune response to the vaccine, so only two doses are needed for full protection.

DeepSeek_2

Docus more frequently presented responses with bullets nested under other bullets or under numbers, but it also occasionally provided responses in paragraph style:

Individuals who experience a life-threatening allergic reaction, such as anaphylaxis, after receiving an HPV vaccine should not continue with the vaccine series. It is crucial to consult a healthcare provider for personalized guidance and to discuss alternative options for protection against HPV-related diseases. Your healthcare provider can assess the situation and provide the best advice tailored to your specific medical needs.

Docus_1

#### Logic and Relevance of Information

All AI chatbots provided logical reasoning to support their answers, often using phrases such as “Here’s why:” [Claude_1, ChatGPT_1, DeepSeek_2, and Docus_1], “Why trust this information?” [ChatGPT_2], and “Why the difference in doses?” [DeepSeek_1]. These explanations were followed with relevant, evidence-based information to justify their responses. For example, when a user asked, “Is the info about the HPV vaccine’s safety even real, or is it just fake?,” AI chatbots offered a clear rationale grounded in scientific research:

7. Why Trust This Information?

The evidence supporting the HPV vaccine’s safety comes from scientific consensus, not just anecdotal reports or single studies. The vaccine has been in use since 2006, and the data from over a decade of global use demonstrates its positive impact on public health.

ChatGPT_2

The HPV vaccine underwent rigorous clinical trials before approval, involving thousands of participants to ensure its safety and efficacy.

Docus_2

#### Details and Length

Some AI chatbots included additional details that, while related, were not directly relevant to the question asked, making their responses unnecessarily long. For instance, when DeepSeek was asked, “Why is the HPV vaccine not recommended after 26 years old?,” it not only explained the main reason but also introduced a new topic: “What Does ‘Shared Clinical Decision-Making’ Mean?” [DeepSeek_2]. Similarly, when asked, “Does the HPV vaccine also protect against other STDs like HIV or chlamydia?,” Docus veered into broader prevention advice, stating: “To reduce the risk of other STDs, including HIV and chlamydia, it’s important to practice safe sexual behaviors, such as...” [Docus_2].

On the other hand, we identified instances where AI chatbot responses did not provide sufficient detail. For example, ChatGPT stated, “Early safety reviews identified rare instances of anaphylaxis, approximately 1.7 cases per million doses, and episodes of syncope (fainting), often related to anxiety” [ChatGPT_1], without clarifying what was meant by “anxiety,” such as fear of needles or an underlying anxiety disorder which could be important for user understanding. Similarly, Claude mentioned, “There’s no evidence of systematic deception about HPV vaccine effectiveness” [Claude_1] but did not provide an explanation of what systematic deception means.

In our sample of 4 AI chatbots, we found that ChatGPT and DeepSeek tended to provide longer and more superfluous responses to questions than Claude and Docus. In this context, superfluous refers to answers that included excessive or unnecessary details and lacked straightforwardness. DeepSeek (510.0 words) had the highest mean word count for responses to each question, while Docus (159.4 words) had the lowest.

#### Conclusion Styles

To conclude their responses to a question, AI chatbots included a summary, follow-up question, further direction, or a combination of these. All AI chatbots frequently provided summaries and often used starting phrases like “The bottom line:” [ChatGPT_1], “In summary” [ChatGPT_2], or “Key takeaways” [DeepSeek_2]. We did not find any responses from DeepSeek or Docus that concluded only with a follow-up question, while ChatGPT and Claude occasionally ended their responses with follow-up questions, such as:

Would you like to dive deeper into any specific studies or concerns about the vaccine?

ChatGPT_1

Would you like more specific information about any of these side effects?

Claude_2

AI chatbots often provided further directions that typically encouraged users to consult a “healthcare provider” [ChatGPT_2, Claude_2, DeepSeek_1, and Docus_2] or refer to trusted sources like the “CDC or WHO” [DeepSeek_1 and Docus_1] to confirm users’ understanding or if users would like to look for further information.

### Linguistic Features

The AI chatbots exhibited diverse linguistic features, from the use of jargon or complex medical terms to providing sentences to foster users’ emotional validation. Not all AI chatbots provided responses tailored to individual users, and emotional validation varied. Some AI chatbots had more understandable responses than others, as evidenced by Flesch-Kincaid scores.

#### Terminology

All AI chatbots occasionally used jargon or medical vocabulary that was infrequently used in lay audience’s everyday speech. Lay users would need to seek out the definitions in order to fully comprehend the provided sentences. Uncommon terminology used included “cervical epithelial neoplasia” and “primary ovarian insufficiency” [ChatGPT_1], “immunosuppressive medications” and “post-market surveillance” [ChatGPT_2], “precancerous lesion” [Claude_1], “vertical transmission” [Claude_2], “salicylic acid or cryotherapy kits” [DeepSeek_1], “fomite transmission” [DeepSeek_2], “anaphylaxis” [Docus_1], and “oropharynx” [Docus_2]. Furthermore, all 4 AI chatbots used the term “gender” when addressing questions on the recipients of the HPV vaccine, despite “sex” being the more precise term in this context, as it pertains to biological distinctions. For instance, Docus remarked:

HPV also affects males and can lead to other types of cancers and genital warts. Therefore, the vaccination recommendations have expanded to include both genders.

Docus_2

#### User Centric and Emotional Validation

DeepSeek and Docus did not provide a user-centric experience, indicated by not incorporating essential users’ personal information in their responses such as age or sex when applicable. Conversely, ChatGPT and Claude incorporated this contextual information. In response to the question, “How many shots do I need to get for the HPV vaccine?,” DeepSeek and Docus offered a broad explanation in correspondence to the age of HPV vaccine initiation and did not provide a personalized reference. ChatGPT provided a specialized segment, “Is it too late to receive the HPV vaccine at 22?” [ChatGPT_1], and Claude stated, “Since you’re 22, you would follow the 3-dose schedule” [Claude_2]. Related to emotional validation, all AI chatbots offered a degree of emotional affirmation, particularly when addressing apprehension around vaccine safety and efficacy. Docus exhibited the least affirming tone for emotional validation, providing only the short “Certainly!” [Docus_1]. Conversely, ChatGPT, Claude, and DeepSeek used more detail and affirming language, likely fostering a sense of acknowledgment among users, for instance:

That’s a great question, and it’s totally understandable to want to be sure about the safety of any vaccine.

ChatGPT_2

It’s normal to have questions about vaccine safety.

Claude_2

It’s understandable why some people might feel like they’ve been lied to about HPV vaccine safety.

DeepSeek_2

Additional quotes related to emotional validation can be found in Multimedia Appendix 3.

#### Relative vs Numeric

All AI chatbots used either relative or exact numerical statistics depending on whether precision was necessary. For instance, when citing specific studies, ChatGPT presented comprehensive numerical data:

Research published in The BMJ highlighted that women vaccinated between ages 12 and 13 showed an 83.9% decrease in cervical cancer rates and a 94.3% reduction in cervical intraepithelial neoplasia grade 3.

ChatGPT_1

In a similar vein, when addressing HPV vaccine recommendations, AI chatbots used precise age ranges and dosage quantities instead of ambiguous phrases such as “teens” or “some doses.” For example:

Two doses: for those starting the vaccine series at age 9-14.

ChatGPT_2

Ages 9-14: Recommended for routine vaccination (2-dose series).

Claude_1

For those aged 9-14: Two doses are recommended, with the second dose given 6-12 months after the first.

DeepSeek_2

For those starting the vaccine series before age 15, two doses are recommended.

Docus_1

Conversely, when precise numbers were unnecessary to understand the context, AI chatbots used relative terminology, such as “nearly,” “some,” or “hundreds,” exemplified by statements like:

In clinical trials, the vaccine was nearly 100% effective at preventing precancerous cervical lesions.

DeepSeek_2

The HPV vaccine has been the subject of hundreds of independent studies from universities, research hospitals, and international health organization.

ChatGPT_1

#### Flesch-Kincaid Scores

**Figure 1:**
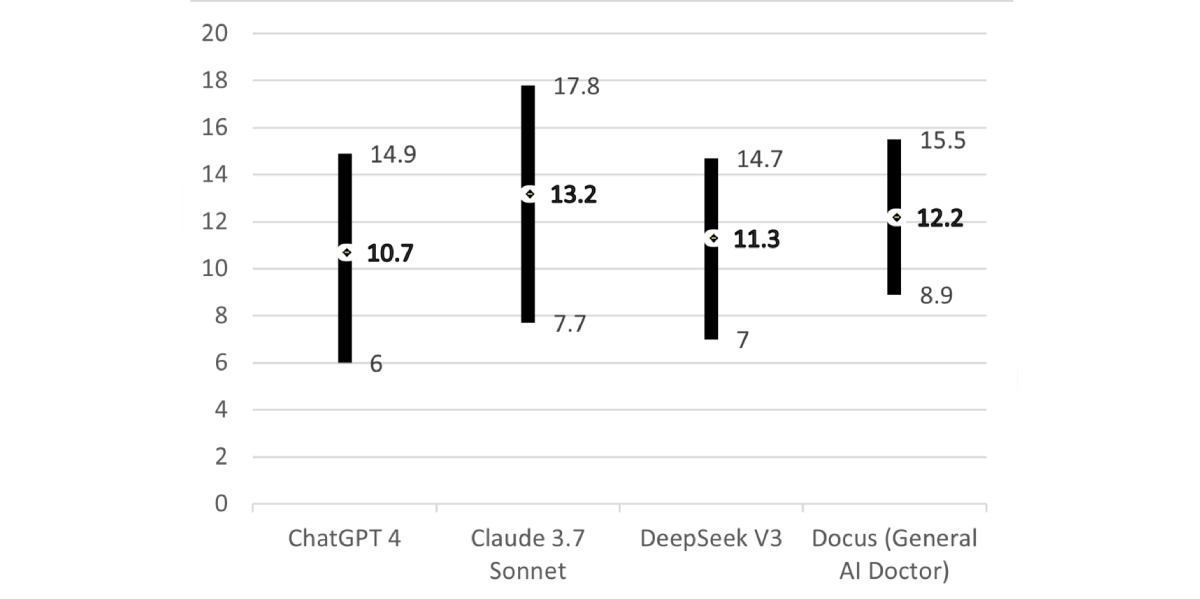
Artificial intelligence chatbots’ Flesch-Kincaid Grade Level (range and mean).

**Figure 2:**
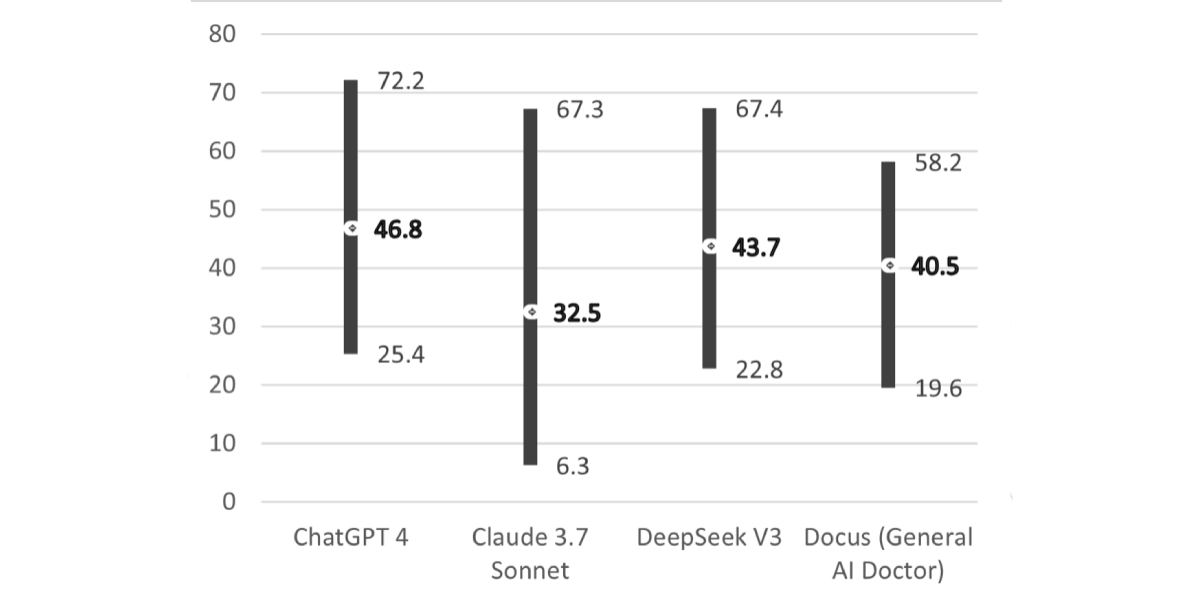
Artificial intelligence chatbots’ Flesch-Kincaid Reading Ease Score (range and mean).

Overall, ChatGPT provided responses with the lowest mean Grade Level and the highest Reading Ease Score among the 4 AI chatbots. In contrast, Claude’s scores had the highest mean Grade Level and lowest mean Reading Ease Score, suggesting that its responses may be more complex than those of the other AI chatbots and potentially less accessible to a lay audience (Figures 1 and 2).

### Accuracy

To examine accuracy, we observed the credibility of links provided in the responses, data accuracy, and intersentence coherence.

#### Information Credibility

When citing links, all AI chatbots included functional links to real, existing websites from reputable organizations, such as the WHO [ChatGPT_1], the American College of Obstetricians and Gynecologists [Claude_1], the European Medicines Agency [DeepSeek_2], and the Centers for Disease Control and Prevention (CDC) [Docus_2]. We found no fabricated or nonexistent links, with no fake studies, fact sheets, or infographics generated. All linked studies were from peer-reviewed academic journals, including the *BMJ* [ChatGPT_1] and the *New England Journal of Medicine* [Docus_1]. However, some of the links provided, particularly those from CDC, were no longer functional at the time of data analysis and led to broken pages displaying “Page Not Found” errors. Furthermore, in a few instances, the AI chatbot responses included links unrelated to the topic asked. For example, when a user requested information about HPV vaccine safety, ChatGPT provided a link to a page on feminizing hormone therapy [70], which contained no information about the HPV vaccine. Similarly, when asked for a study on HPV vaccine safety, Docus linked to a measles vaccine study [71], which also had no relevance to the HPV vaccine (Table 2).

**Table 2:** Comparison of 4 artificial intelligence (AI) chatbot responses based on selected measurements.

Chatbots		ChatGPT 4	Claude 3.7 Sonnet	DeepSeek V3	Docus (General AI Doctor)
**Structure and pattern**					
	Organization format^a^	Bulleted points nested under numbered list	Bulleted points	Bulleted points nested under numbered list	Bulleted points nested under bulleted points
	Word counts per question, mean (range)	436.4 (218-954)	188.0 (138-255)	510.0 (325-735)	159.4 (61-200)
	Conclusion styles^b^	Summary and further direction	Summary and further direction	Summary and further direction	Further direction
**Linguistic features**					
	Jargon/medical terminology^c^	Immunosuppressive medications, postmarket surveillance	Precancerous lesion prevention, vertical transmission	Cryotherapy kits, fomite transmission	Anaphylaxis, oropharynx
	Personalization^d^	Yes	Yes	No	No
	Emotional validation^e^	“That’s a great question, and it’s totally understandable to want to be sure about the safety of any vaccine.”	“It’s normal to have questions about vaccine safety.”	“It’s understandable why some people might feel like they’ve been lied to about HPV vaccine safety.”	“Certainly!”
**Accuracy**					
	Links credibility^f^	Yes	Yes	Yes	Yes
	Fabricated sources^g^	No	No	No	No
	Incorrect links^h^	Yes	No	No	Yes
	Statistics accuracy^i^	Yes	Yes	Yes	Yes
Currency^j^		No	Yes	Yes	Yes
Vaccination stance^k^		Pro	Neutral	Pro	Pro

^a^Most frequently used response structure to a question (eg, using bullet points, numbered lists, paragraphs, or combinations).

^b^Most frequent way responses ended to each question (eg, with summary, follow-up questions, further suggestion, or combinations).

^c^Example of jargon/medical terminology used.

^d^Referenced user’s sex and ages when necessary.

^e^Example of affirming language used to acknowledge user’s feeling.

^f^Always provided real link from reputable organization or peer-reviewed journal.

^g^Ever provided link/information that are made up or nonexistent.

^h^Ever provided links that are not related to the discussed topic.

^i^Always provided/cited accurate statistic numbers based on scientific evidence.

^j^Always provided information or fact sheets that were not older than 12 months or studies not older than 5 years.

^k^Most frequent stance on HPV vaccination per question (eg, contra, neutral, or pro).

#### Data Accuracy and Coherence

We found no inaccuracies in the numerical data or statistics provided by the AI chatbots in our sample. Additionally, when responses included links to studies, the statistical values cited in the responses matched those in the referenced sources. However, we did identify an instance of internal inconsistency in Claude’s and DeepSeek’s responses. Claude initially stated, “Based on extensive safety monitoring and research, there aren’t specific demographic groups who systematically experience significantly more problems with HPV vaccines” [Claude_2]. Yet, it immediately followed with, “However, there are a few considerations worth noting:” and listed groups who should avoid the vaccine, such as individuals with yeast allergies. Similarly, DeepSeek mentioned, “There are currently three vaccines available: 1. Gardasil 9: Protects against 9 types of HPV (6, 11, 16, 18, 31, 33, 45, 52, and 58). It is the most commonly used vaccine in many countries” [DeepSeek_2], but only mentioned Gardasil 9 and failed to elaborate on the other two vaccines referenced in the previous sentence or specify that they are not all available worldwide. For example, only Gardasil 9 is currently available in the United States.

### Currency

Some of the studies cited by ChatGPT were more than 5 years old when addressing whether the HPV vaccine affects fertility. While these studies were not outdated, we applied a 5-year cutoff as a point of comparison to assess the currency. For example, ChatGPT referenced, “A 2018 study in Pediatrics” and “a 2019 Australian study” [ChatGPT_1]. However, we did not find any information or fact sheets that were older than 12 months or studies older than 5 years in responses from the other AI chatbots.

### HPV Vaccination Stance

Across all responses, none of the AI chatbots expressed opposition to HPV vaccination, as the tone remained either neutral or supportive. Even when asked whether individuals with a history of life-threatening allergic reactions to the HPV vaccine should get vaccinated, none of the AI chatbots directly advised against it. Instead, they consistently recommended consulting health care professionals. Examples include:

Talk to an allergist or immunologist before getting vaccinated.

ChatGPT_1

Your healthcare provider can help assess the specific situation and determine whether it was truly a severe allergic reaction or another type of side effect that might not prevent further vaccination.

Claude_1

...the decision to receive the HPV vaccine requires careful consideration and consultation with a healthcare provider.

DeepSeek_1

Your healthcare provider can assess the situation and provide the best advice tailored to your specific medical needs.

Docus_1

## Discussion

### Principal Findings

Our study examined the response of 4 AI chatbots (ChatGPT 4, Claude 3.7 Sonnet, DeepSeek V3, and Docus [General AI Doctor]) in answering HPV vaccine–related questions, with an emphasis on structure and patterns, linguistic features, accuracy, information currency, and stance on HPV vaccination. Our results showed that while all AI chatbots responded based on scientific research and reputable websites, there were some variations in response length, details, readability, and personalization. All of the AI chatbots presented valid scientific references without fabrication of sources, but there were instances of AI chatbots providing broken or irrelevant links. While statistical data were accurate for all AI chatbots, several showed inconsistencies across sentences and lack of informational completeness. Although the AI chatbots did not disseminate incorrect information, their utility in public health communication is still mixed in effectively providing information about HPV vaccination in a young adult population.

Our AI chatbots sample provided responses based on scientific data, cited websites of reputable organizations, and displayed no evidence of falsification of sources. These findings imply that AI chatbots can offer factually accurate information on the HPV vaccine, similar to recent studies that discussed AI chatbots’ inclusion of accurate medical information in their responses [43,72]. A systematic review also highlighted that AI chatbots have primarily been used to deliver factual information in response to users’ vaccine-related questions [73]. However, when we verified the links provided in the responses, we occasionally discovered broken or irrelevant links. The majority of the broken links came from the CDC website, which may have been restructured in accordance with directives from the current administration [74,75]. Consequently, certain links that were operational at the time of data collection may have become unreachable during later analysis. As public health information is often shaped by political leadership, static AI chatbot content may quickly become outdated if it is not updated rapidly. Despite finding fewer than 5 irrelevant links, this instance may increase apprehension regarding the credibility of these AI chatbots as a medium to disseminate health information. Although these irrelevant links were not fabricated, as is often a concern in studies examining AI chatbot “hallucination” where responses contain made-up references that do not correspond to any existing studies [76,77], they still pose challenges. Users who attempt to verify information through these links may experience reduced trust or increased frustration when attempting to make informed health decisions.

The readability of AI chatbot responses in our sample, assessed using Flesch-Kincaid’s Grade Level and Ease Score, would be challenging for a lay audience to comprehend. The mean Grade Level scores for each AI chatbot ranged from 10.7 to 13.2 (ChatGPT: 10.7; Claude: 13.2; DeepSeek: 11.3; and Docus: 12.2). The Reading Ease Score was 32.5 to 46.8 (ChatGPT: 46.8; Claude: 32.5; DeepSeek: 43.7; and Docus: 40.5). Therefore, ChatGPT was the most comprehensible and Claude the least comprehensible in each Flesch-Kincaid measure. Nevertheless, all AI chatbot means of Grade Level were beyond 8 and the Reading Ease Scores were below 50, indicating that all AI chatbots in our sample were deemed difficult for a lay audience to comprehend [49]. A recent study on AI chatbots also reported a high mean of Grade level score and a low mean of Reading Ease score [51]. This is concerning, as health information that exceeded suggested readability levels may create barriers to comprehension for persons with lower health literacy [78]. Additionally, the prevalent use of medical jargon among all AI chatbots in our sample may exacerbate the readability issue. Many lay audiences without medical backgrounds may not be familiar with medical terminologies such as “cervical epithelial neoplasia” and “immunosuppressive medications.” Without providing definitions or further explanation of these terminologies, it could further impede understanding. Moreover, the excessive length of responses from ChatGPT and DeepSeek may overwhelm users. Recent studies indicated that information overload, described as a condition in which an individual is unable to comprehend or respond to stimuli due to excessive information [79], may counterproductively reduce the ability to process information [80] and eventually affect HPV vaccination decision-making. In light of these challenges revealed by our study, AI developers should explore AI chatbot optimization approaches to improve readability for lay audiences, by implementing features like real-time readability adjustment, automatic jargon detection, and using summary-first formats that provide essential points up front, with optional details available on demand.

The degree of personalized responses varied across AI chatbots in our sample, with ChatGPT and Claude often referencing user characteristics such as age and sex in their responses, while DeepSeek and Docus did not, despite these user characteristics being provided at the beginning of the conversation. Personalization in health communication has been shown to enhance user experience by increasing perceived benefits and self-efficacy [81]. When AI chatbots did not include personalization, they may have missed opportunities to deliver tailored information that could have enhanced the uptake of HPV vaccination. In addition to personalization, using affirming language that acknowledges user concerns or feelings can further improve user experience and eventually improve engagement, particularly when discussing stigmatized topics like sexually transmitted infections caused by HPV [82]. However, it is important to note that implementing personalization and affirming language may require AI chatbots to retain user information, sometimes without the user’s explicit consent, which in turn raises concerns about data privacy and security [83,84]. A study found that 47% of the respondents were concerned about the security of information entered into AI chatbots, which may reduce AI chatbots' acceptability in health care services [27]. To minimize these risks, robust data protection protocols are essential to safeguard user privacy when interacting with AI chatbots [83].

The uniformly neutral or provaccine stance exhibited by all AI chatbots in our sample corresponds with the scientific consensus, even when asked questions rooted in vaccination conspiracy beliefs. We found that AI chatbots maintained evidence-based stances without embracing antivaccine rhetoric. If AI chatbots take an antivaccine stance, it can fuel misinformation, such as antivaccine statements are scientifically unconfirmed and often rooted in personal stories [10,85]. While neutrality may not directly challenge misinformation, it offers an opportunity to present information in a nonjudgmental manner without using strongly persuasive messaging, helping to build trust among vaccine-hesitant individuals [86,87]. In challenging questions where AI chatbots were questioned about obtaining a vaccine despite potential life-threatening reactions, AI chatbots typically deferred to health care providers for evaluation rather than becoming the decision maker. By guiding users to health care professionals, AI chatbots effectively position themselves as supportive tools rather than substitutes for health care providers [88]. Chatbots can enhance online interactions between patients and health care professionals, especially when navigating complex medical questions [89].

### Limitations

This study has multiple limitations. First, our data collection was performed during a defined period (February 22-28, 2025), documenting AI chatbot responses at a singular timeframe of their evolution. Due to the rapid advancement of AI technology, these findings may not accurately represent the current capabilities of these platforms. Second, our analysis focused on text-based responses and did not assess multimedia components that could support information dissemination on certain AI chatbots. Third, there was a time lapse of several weeks between data collection and data analysis, during which some of the links provided in AI chatbot responses were no longer available due to shifts in political leadership. Fourth, while we simulated young adult users, actual interactions may differ based on user’s specific characteristics and differences in query formulation. Furthermore, it may not fully capture the diversity and natural language patterns seen in actual queries from young adults. Our question set may also not fully capture the breadth of real-world HPV-related concerns among young adults, although its development based on Google Trends data may reflect what users are commonly searching for in an online space. Furthermore, we did not measure actual user comprehension or behavioral outcomes following interactions with these AI chatbots, limiting our ability to assess their real-world relationship on HPV vaccination decisions. Fifth, we used AI chatbots with different update methods (static and dynamic) that may partially explain why some AI chatbots provided less relevant information. Finally, the generalizability of our findings may be limited as our sample focused on a subset of AI chatbots mimicking young adult users in the United States. We analyzed 2 queries per AI chatbot, which limits the ability to fully assess response variability and consistency within each platform. We focused on a select group of AI chatbots based on some sets of criteria, not comprehensively including more AI chatbots. Therefore, results may not extend to other populations, age groups, or other AI chatbots not assessed in this study.

### Conclusions and Recommendations

Our study highlights the potential of AI chatbots as sources of generally accurate HPV vaccine information for young adults, potentially serving as alternatives to the misinformation-rich health content often found online. However, challenges remain in verifying the sources or links provided, ensuring readability, and maintaining personalization. These challenges underscore the urgent need for the development and adoption of standardized evaluation tools to ensure the efficacy of AI chatbots when they are used to supply health information to lay audiences. Such standardized tools would enable consistent benchmarking of AI chatbots' performance and facilitate meaningful comparisons in their ability to deliver reliable health information to lay audiences. In addition, standardized tools could help identify gaps in AI chatbot knowledge and functionality, guiding developers in refining these systems. To ensure information remains current and reliable, AI chatbots should implement regular knowledge updates and systematic checks for source accuracy. To improve readability, AI chatbots should explore optimization approaches such as real-time readability adjustment. To enhance personalization, developers should design AI chatbot responses that are tailored to users while safeguarding their data privacy and security.

Our findings also signal the emergence of fostering “AI health literacy,” which refers to the ability of individuals to understand and verify health information generated by AI tools. Moving forward, public health interventions must focus not only on the supply side by improving AI chatbots technology, but also on the demand side by equipping users with skills to verify AI-generated content to mitigate the overreliance on misinformation. We also recommend that users appropriately position AI chatbots as complements to, rather than replacements for, health care professionals’ guidance. Given that AI chatbots sometimes adopt a neutral stance when discussing HPV vaccination, understanding the implications of this neutrality for combating misinformation and promoting vaccine confidence remains an important direction for future research.

Finally, moving forward, health policies should focus on partnering with technology firms to establish a systematic process for AI chatbots to help users distinguish high-quality and low-quality AI chatbots used in health care services. By addressing these considerations, AI chatbots could become accessible and valuable tools for improving HPV vaccine knowledge and potentially increasing vaccination rates among young adults.

## Appendix

Multimedia Appendix 1 Questions set.

Multimedia Appendix 2 Study codebook.

Multimedia Appendix 3 Additional quotes.

## Abbreviations

AI: artificial intelligence
CDC: Centers for Disease Control and Prevention
COREQ: Consolidated Criteria for Reporting Qualitative Research
HPV: human papillomavirus
LLM: large language model
VCBS: Vaccine Conspiracy Beliefs Scale
WHO: World Health Organization

## Data Availability

The datasets used and analyzed during the study are available from the corresponding author on reasonable request.
