# Supplementary material for: Examining Artificial Intelligence Chatbots’ Responses in Providing Human Papillomavirus Vaccine Information for Young Adults: Qualitative Content Analysis
Source: JMIR Public Health Surveill. 2026 Feb 18;12:e79720. doi: 10.2196/79720 (PMC12961391; doi:10.2196/79720)
Supplement: Multimedia Appendix 1 [file publichealth_v12i1e79720_app1.pdf]

	Questions	Original wording
0	I am 22 year old (woman/man) looking for information about the human papillomavirus vaccine. Can you help me?	
**Vaccine Conspiracy Beliefs Scale Adaptation**		
1	Is the info about the HPV vaccine's safety even real, or is it just fake?	Vaccine safety data is often fabricated.
	1a. Where can I actually get reliable info about the HPV vaccine safety?	
2	Are the harms of the HPV shot covered up?	Immunizing children is harmful and this fact is covered up.
	2a. What are the side effects after getting HPV shots?	
	2b. Do certain groups of people have more problems after getting HPV shots?	
3	Are drug companies covering up how dangerous the HPV vaccine might be?	Pharmaceutical companies cover up the dangers of vaccines.
4	Are people lied to about how well the HPV vaccine works?	People are deceived about vaccine efficacy
5	Do companies make up the data about how well the HPV vaccine works?	Vaccine efficacy data is often fabricated.
6	Are people lied to about HPV vaccine safety?	People are deceived about vaccine safety
	6a. Why does it seem like some people believe they've been lied to about HPV vaccine safety?	
7	Is the government hiding the link between HPV vaccine and infertility?	The government is trying to cover up the link between vaccines and autism.
**Google Trends Rising Queries Adaptation (Jan 2024-25)**		
8	How many shots do I need to get for the HPV vaccine?	hpv vaccine 3-dose schedule for adults
	8a. If I already have HPV, will the shot get rid of it?	
9	Can you get HPV without having sex?	can you get hpv without having sex
10	Why is HPV vaccine not recommended after 26 years old?	why is hpv vaccine not recommended after 26
**Google Trends Top Queries Adaptation (Jan 2024-25)**		
11	What age is the HPV vaccine recommended for?	hpv vaccine age
12	What's the HPV vaccine for?	what is hpv vaccine
13	Does the HPV vaccine prevent all warts?	hpv warts
	13a. Does the HPV vaccine also protect against other STDs like HIV or chlamydia?	
14	Why is the HPV vaccine only recommended for women?	hpv vaccine men
	14a. Why do men need the HPV vaccine if they can't get cervical cancer?	
