# Supplementary material for: Examining Artificial Intelligence Chatbots’ Responses in Providing Human Papillomavirus Vaccine Information for Young Adults: Qualitative Content Analysis
Source: JMIR Public Health Surveill. 2026 Feb 18;12:e79720. doi: 10.2196/79720 (PMC12961391; doi:10.2196/79720)
Supplement: Multimedia Appendix 2 [file publichealth_v12i1e79720_app2.pdf]

CHATBOT STUDY CODEBOOK

PATTERN

- Ended with questions
- Ended with conclusion
- Ended with further direction
- Ended with conclusion and further direction
- Ended with others
- Answer logic
- Relevant information
- Irrelevant information
- Counter evidence
- Bold text
- Paragraph
- Bullet point
- Numbered point

COMMUNICATION

- Jargon
- Flesch-Kincaid_Grade level
- Flesch-Kincaid_Ease score
- Lack of coherency
- Emotional validation
- User-Centric
- Superfluous
- Succinct
- Lack of Details
- Relative
- Numeric

ACCURACY

- Real website
- Not found website
- Incorrect website
- Real study
- Reliable website
- Reliable study
- Inaccurate statistics
- Inaccurate information
- Numbers not in source
- Unsuitable term

LATEST INFORMATION

- Up-to-date information
- Out-of-date information
-

VACCINE STANCE

- Pro
- Contra
- Neutral
