# Supplementary material for: Examining Artificial Intelligence Chatbots’ Responses in Providing Human Papillomavirus Vaccine Information for Young Adults: Qualitative Content Analysis
Source: JMIR Public Health Surveill. 2026 Feb 18;12:e79720. doi: 10.2196/79720 (PMC12961391; doi:10.2196/79720)
Supplement: Multimedia Appendix 3 [file publichealth_v12i1e79720_app3.docx]

Table 3. Emotional Validation Quotes from Artificial Intelligence Chatbots’ Responses

| **Chatbot** | **Question** | **Response** |
| --- | --- | --- |
| ChatGPT | Do certain groups of people have more problems after getting HPV shots? | “That's a thoughtful question—**certain groups may experience different reactions** to the HPV vaccine, but ….” |
|  | Are drug companies covering up how dangerous the HPV vaccine might be? | “I get why these doubts might stick around, especially with valid concerns about the pharmaceutical industry.” |
|  | Do companies make up the data about how well the HPV vaccine works? | “It’s completely valid to wonder if companies might manipulate data” |
|  | Is the info about the HPV vaccine’s safety even real, or is it just fake? | “That’s a great question, and it’s totally understandable to want to be sure about the safety of any vaccine.” |
|  | Where can I actually get reliable info about the HPV vaccine safety? | “It’s great that you’re looking for reliable sources to get accurate information” |
| Claude | Is the info about the HPV vaccine’s safety even real, or is it just fake? | “It's normal to have questions about vaccine safety.” |
| Deepseek | I am 22 year old woman looking for information about the human papillomavirus vaccine. Can you help me? | “Absolutely, I’d be happy to help you with information about the human papillomavirus (HPV) vaccine.” |
|  | Why does it seem like some people believe they've been lied to about HPV vaccine safety? | “It’s understandable why some people might feel like they’ve been lied to about HPV vaccine safety, even though the scientific evidence overwhelmingly supports its safety and effectiveness.” |
| Docus | Can you give me some sources from peer-reviewed publications? | “Certainly! Here are some peer-reviewed publications that provide…” |
|  | I am 22 year old woman looking for information about the human papillomavirus vaccine. Can you help me? | “Certainly! The Human Papillomavirus (HPV) vaccine is …” |
